# Batch and Continuous Lactic Acid Fermentation Based on A Multi-Substrate Approach

**DOI:** 10.3390/microorganisms8071084

**Published:** 2020-07-21

**Authors:** Agata Olszewska-Widdrat, Maria Alexandri, José Pablo López-Gómez, Roland Schneider, Joachim Venus

**Affiliations:** Department of Bioengineering, Leibniz Institute for Agricultural Engineering and Bioeconomy, Max-Eyth-Allee 100, 14469 Potsdam, Germany; malexandri@atb-potsdam.de (M.A.); plopezgomez@atb-potsdam.de (J.P.L.-G.); rschneider@atb-potsdam.de (R.S.)

**Keywords:** lactic acid, continuous fermentation, multi substrate fermentation

## Abstract

The utilisation of waste materials and industrial residues became a priority within the bioeconomy concept and the production of biobased chemicals. The aim of this study was to evaluate the feasibility to continuously produce L-lactic acid from different renewable substrates, in a multi-substrate strategy mode. Based on batch experiments observations, *Bacillus coagulans* A534 strain was able to continuously metabolise acid whey, sugar beet molasses, sugar bread, alfalfa press green juice and tapioca starch. Additionally, reference experiments showed its behaviour in standard medium. Continuous fermentations indicated that the highest productivity was achieved when molasses was employed with a value of 10.34 g·L^−1^·h^−1^, while the lactic acid to sugar conversion yield was 0.86 g·g^−1^. This study demonstrated that LA can be efficiently produced in continuous mode regardless the substrate, which is a huge advantage in comparison to other platform chemicals.

## 1. Introduction

The vast majority of lactic acid (LA) produced in the world comes from fermentation processes. Typically, simple sugars such as glucose and sucrose, obtained mainly from corn, sugarcane, and cassava [1], are used as substrates in LA fermentations. However, the introduction of new concepts, such as biorefinery and circular bioeconomy, have pushed towards the utilisation of other raw materials [2]. The search for alternative cheaper substrates not only aims to cut production costs, but also to implement better disposal and management systems as, in most cases, the new substrates researched are by-products or waste streams from other processes [3,4]. 

Several alternative substrates have been investigated to produce LA such as garden wastes [5], food wastes [6], organic municipal solid wastes [7,8] and lignocellulosic wastes [9,10] to name a few. However, in comparison to processes which use simple sugars, the valorisation of this kind of substrates comprises some extra challenges. First, they are complex and heterogeneous materials which normally require a pretreatment to make sugars available for microbial growth. Additionally, unlike current industrial fermentations in which a single simple sugar is used, fermentation media from alternative substrates usually contain a combination of carbohydrates. Therefore, microbes able to metabolise different sugars, ideally simultaneously, are of high interest. Several reports concerning the utilization of various sugars in LA fermentations such as glucose, galactose, fructose, etc., separately or a mixture of them, are available in the literature [11,12,13,14,15]. Although insightful, in most cases experimental work involved pure sugars that do not replicate the conditions of complex substrates. 

Another challenge for the utilisation of inexpensive substrates can be that of supply. Thus, even though the processes can prove to be feasible from a biochemical perspective, the available quantities of substrate can be a constraint. For instance, several of the substrates which could be used in LA fermentation are influenced by seasonality, e.g., agro-industrial residues [16]. Evidently, a LA production plant cannot function intermittently throughout the year. A potential solution for this problem could be a system in which LA is produced continuously from diverse substrates. However, the development of efficient bioprocesses is highly dependent on the applied feedstock, and variations of the initial raw material could lead to lower yields and productivities. Fermentations with mixed cultures have been tested to overcome the heterogeneity problems [17,18,19,20]. Nonetheless, although using mixed populations of bacteria can bring benefits, the control and maintenance of operational conditions to achieve high efficiencies is a burden. An alternative simpler solution might be a system based on the utilisation of a robust microorganism, able to withstand substrate variations while maintaining high efficiencies. 

*Bacillus coagulans* spp. is known to have good environmental tolerance, low by-product formation and high conversion efficiencies [21], and their effectiveness in the bioconversion of a wide number of substrates has already been shown [5,22,23,24,25,26,27]. In addition, *B. coagulans* strains can homofermentatively transform various types of sugars including glucose, xylose, lactose, etc. into L-LA. Furthermore, with optimal temperatures of growth between 50 and 55 °C, fermentations by *B. coagulans* allow to carry out non-sterile processes with a reduced risk of contamination and comparable costs to fermentations at lower temperatures [28]. 

This article reports the results obtained using *B. coagulans* for the fermentations of molasses, alfalfa juice, acid whey, sugar bread and tapioca starch hydrolysates, in batch and continuous mode.

## 2. Materials and Methods

### 2.1. Substrates

Acid whey was supplied by Glanbia Ingredients, Marian Lacey, Ballyragget (Kilkenny, Ireland). Sugar beet molasses were purchased by Pfeifer & Langen GmbH & Co., KG (Köln, Germany). Sugar bread was kindly provided by CETECE Centro Tecnológico (Palencia, Spain). Alfalfa press green juice was purchased from Leibniz-Zentrum für Agrarlandschaftsforschung (ZALF) and pressed at ATB. Tapioca starch was supplied by Emsland Stärke GmbH (Emlichheim, Germany). The composition of all substrates is presented in Table 1.

### 2.2. Microorganism Used and Inoculum Preparation

*Bacillus coagulans* A534 strain was used for the whole set of experiments, both batch and continuous fermentations, using acid whey, molasses, alfalfa green juice, tapioca starch and sugar bread hydrolysates. The inoculum was prepared in MRS broth (Merck, Germany) with dolomite EVERZIT Dol (0.5–2.5 mm (Evers, Germany) as buffering system, at an orbital shaker set at 100 rpm, 52 °C for 14 h. Inocula for the fermentations were prepared in MRS medium as described elsewhere [29]. The number of cells added to each batch fermentation was determined using THOMA cell chamber and the average cell amount reached 0.99 × 10^12^ of total cells/L. The number of living cells, given as colony-forming unit (cfu), was monitored by making sequential dilutions. One-hundred microliters was pipetted onto a petri dish filled with Nutrient Agar (Merck, Germany). After 24 h incubation at 52 °C, the number of colonies was calculated.

Measurement of dry cell weight (DCW) was applied in order to determine the microbial growth. The collected sample pellet was washed with demineralised water, centrifuged at 5000 rpm, at 4 °C, for 15 min. Dry cell weight measurements were carried out by drying the cell biomass at 105 °C until constant weight. The average value of two-fold weighting was calculated.

### 2.3. Substrate Preparation

Acid whey was sterilised at 121 °C for 15 min. Yeast extract was autoclaved separately, under the same conditions. Substrate was firstly microfiltered through 0.2 µm ceramic membranes (Inside Cèram) and the remaining retentate was then diluted in water, at a ratio 40:60 and autoclaved at 121 C for 15 min.

The molasses containing 240 g·L^−1^ of total sugars were diluted in water to a final sugar concentration of 120 g·L^−1^ and autoclaved at 121 °C for 15 min.

Sugar bread hydrolysis was carried out in two steps (liquefaction and saccharification) at 72 L BIOSTAT Bplus (B-Braun Biotech, Hessen, Germany), with 60 L working volume. A suspension of sugar bread in water (15.5% *w*/*v*) was added to the bioreactor under unsterile conditions. The pH was set to a value of 6.0 with 20 % (*w*/*v*) NaOH. Liquefaction was carried out at 80 °C, using 4.4 mL of the enzymatic preparation BAN 240 L (Novozyme), under constant stirring (300 rpm) for 2 h. The saccharification step was performed at 52 °C, at pH 4.5 adjusted with 20% NaOH (*w*/*v*). The enzymatic preparation Stargen 002 (Fa. Genencor) was subsequently added at a dosage of 1 mL per L of substrate at a stirring rate of 300 rpm, which was switched to 200 rpm by the end of saccharification. At the end of the hydrolysis (25 h in total), the medium was collected and microfiltered through TAMI 0.2 µm ceramic membranes (Inside Cèram).

Alfalafa green juice was microfiltered through 0.2 µm ceramic membranes (Inside Cèram) and sterilised at 121 °C for 15 min.

Tapioca hydrolysis was done in the same way as sugar bread, but with 55 L working volume. Tapioca starch was diluted in water (14.3% *w*/*v*) and added to the bioreactor under unsterile conditions. After 20 h, the solution was ultra-filtered with 0.1 µm ceramic membranes and used for the continuous fermentations.

Different batches were tested in order to monitor the performance of the strain in separated experiments. This information was necessary before continuous mode was applied to be sure that *B. coagulans* A534 is able to utilise each substrate.

### 2.4. Fermentation

Batch fermentations were carried out in 2 L bench-top bioreactors (BIOSTAT Bplus 2), with 1 L working volume. Experiments were performed at 52 °C, with stirring speed at 200 rpm, at a pH of 6.5, adjusted via 20% NaOH solution, in the presence of 15 g·L^−1^ yeast extract. Reference fermentation was carried out in 1 L bioreactor containing a mixture of 10 g·L^−1^ glucose, fructose, galactose, xylose, arabinose, lactose, sucrose and maltose each. Additionally, to support the growth, yeast extract was added (15 g·L^−1^).

Two continuous fermentations were carried out in 5 L BIOSTAT Bplus3 bioreactors (Sartorius AG, Germany), with 3 L working volume, using a cell retention module with hollow-fibre filters UMP—1047R (Pall Corporation). The experiments were performed at 52 °C, with stirring at 300 rpm, at a pH of 6.5, adjusted via 20% NaOH solution, in the presence of 30 g·L^−1^ yeast extract. The first continuous fermentation was carried out with acid whey, molasses and tapioca, whereas the second continuous fermentation was carried out with acid whey, molasses, sugar bread and alfalfa green juice. Alfalfa green juice was used as a substitute for yeast extract in order to evaluate it as a cheaper nitrogen source. 

The continuous mode was operated under a constant dilution rate of 0.2 h^−1^. Two feeding solutions were used: feed 1 containing a given substrate and feed 2 containing 30 g·L^−1^ yeast extract. The carbon and nitrogen source were fed to the reactor at a ratio of 50:50. The same strategy was followed also when alfalfa green juice was used as substitute to yeast extract. In that case, sugar bread and green juice were fed in the bioreactor at a ratio of 50:50.

Productivities were calculated as the global productivity (Pg) and the maximum productivity (Pmax). The global productivity (Pg) was the productivity calculated from the beginning of fermentation, the moment of inoculation, until the beginning of stationary phase, where LA production was finished. Productivities were calculated at every sampling point and the maximum productivity (Pmax) was the highest value calculated. For the continuous mode global productivity was calculated as the multiplication of LA concentration in each sampling point and dilution rate (D = 0.2 h^−1^). Afterwards the average was shown.

### 2.5. Analytical Methods

Sugar content and lactic acid concentration were measured via HPLC (DIONEX, Sunnyvale, CA, USA), connected with a refractive index detector (RI-71, Shodex, Yokohama, Japan) and supplied with a Eurokat H column (300 mm × 8 mm × 10 µm, Knauer, Berlin, Germany), eluted with 5 mM H_2_SO_4_ at a flow rate of 0.8 mL·min^−1^. The analysis of cations in the substrates, hydrolysis and fermentation samples was done via an IonPac CS 16 column (250 mm × 4 µm, DIONEX, Sunnyvale, CA, USA), operating at a flow rate of 1.0 mL·min^−1^, at 40 °C, with 30 mM CH_3_SO_3_H as mobile phase. An IonPac As9-HC column (250 mm × 4 µm, DIONEX, Sunnyvale, CA, USA) was used for the analysis of anions, eluted with Na_2_CO_3_ at a flow rate of 1.2 mL·min^−1^, at room temperature.

Lactic acid optical purity analysis was done using HPLC (Knauer, Berlin, Germany) coupled with a Chiralpak^®^MA(+) column (Daicel, Tokyo, Japan, 50 mm × 4.6 mm × 3 µm), where 2 mM CuSO_4_ was used as mobile phase at a flow rate of 0.8 mL·min^−1^, coupled with an ultraviolet detector. Protein content and the determination of total phosphorus (P) content were measured following the standard method [29] and via flow injection analysis (FIA), according to the international standard [30].

## 3. Results

### 3.1. Substrate Composition

The composition of substrates before and after the pretreatment is shown in Table 1 and Table 2, respectively.

Acid whey was mostly composed of lactose with a concentration of 419 g·L^−1^, but other disaccharides were also detected. It also contained fructose and galactose, followed by lactic acid, nitrogen and phosphorus. Molasses comprised mostly of disaccharides (sucrose), but glucose and fructose were also detected. The concentration of LA measured was similar to the concentration of LA in whey (~30 g·L^−1^). Moreover, nitrogen was observed and small amount of phosphorus. Sugar bread contained disaccharides (sucrose), glucose and a small amount of fructose. Nitrogen and phosphorus were also detected. A low amount of disaccharides (1.42 g·L^−1^) was detected in alfalfa green juice as well as fructose and glucose, whereas tapioca consisted mostly of starch (95% of its DW), but also contained 0.022% of nitrogen (Table 1).

Only sugar bread and tapioca needed additional enzymatic pretreatment in order to release sugars from starch. Acid whey, molasses, and alfalfa juice were filtered and autoclaved. Their composition after the pretreatment is shown in Table 2.

Membrane filtration is commonly used for acid whey pretreatment, especially for LA removal. As shown in Table 2, LA concentration was reduced by 75%, which is quite important for both LA fermentation and purification. This value is consistent with other literature cited-publications. A combination of nanofiltration and diafiltration led to a removal of 66% of LA from acid whey [31]. In the work of Chen et al. [32], approximately 80% of LA was separated from acid whey by ultrafiltration and electrodialysis. At the same time, no significant losses on sugars and other nutrients was detected.

Starch hydrolysis of sugar bread and tapioca led to a release of almost 73 g·L^−1^ and 141.6 g·L^−1^ of glucose, respectively (Table 2). After hydrolysis an amount of 10 g·L^−1^ of fructose was also detected in sugar bread hydrolysate, whereas disaccharide concentration increased to 38.5 g·L^−1^. This could be most probably attributed to the presence of maltose. However, due to analytical restrictions, it was not possible to measure sucrose and maltose, separately.

### 3.2. Batch Fermentations

**Figure 1:**
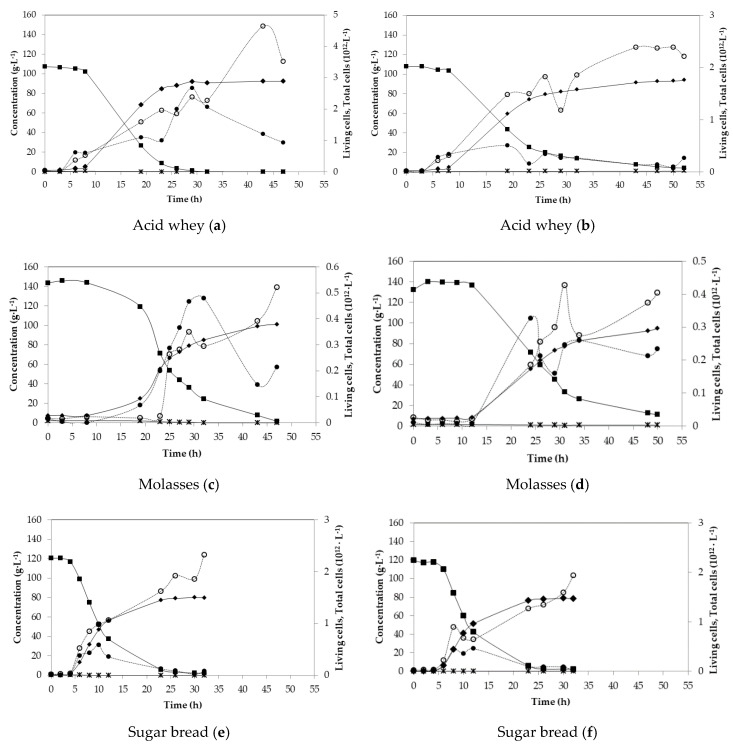

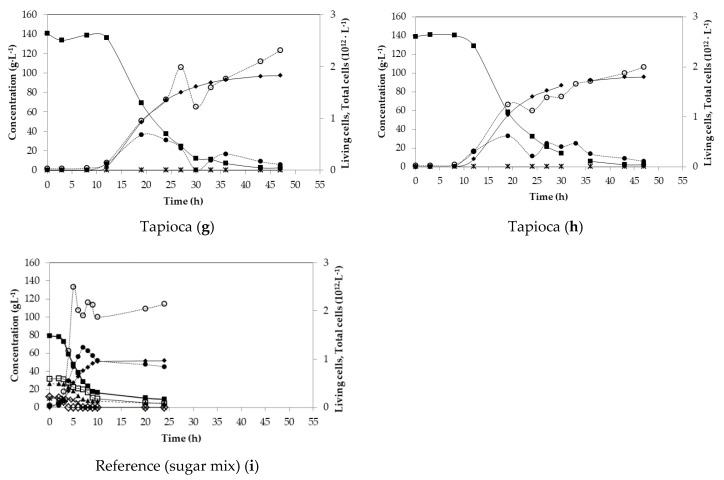
Batch fermentations of acid whey (**a**,**b**), molasses (**c**,**d**), sugar bread (**e**,**f**) and tapioca (**g**,**h**), with standard medium (**i**) using *B*. coagulans A534 strain. Concentration in g·L^−1^ of total sugars (closed squares), glucose (open diamonds), lactose (open squares), galactose (triangles), arabinose (crosses) consumption, acetic acid (stars) and lactic acid (closed diamonds) production. Measurements of living and total cells per L. Living cells (closed circles) and total cells (open circles).

Lab-scale fermentations were carried out in order to optimise the fermentation process and monitor the behaviour of *B. coagulans* A534 strain. Figure 1 shows the fermentation profiles for all substrates, as duplicates. Initial sugar content with respect to tested substrates ranged from 107 g·L^−1^ for acid whey, up to 143 g·L^−1^ of total sugars for molasses. The distribution of sugars in duplicate experiments was similar for each substrate. However, sugar consumption was different (Figure 1). In the case of acid whey, in the first batch, sugars were consumed after 29 h, but in the second fermentation, after 52 h, there was still 4.24 g·L^−1^ of unconsumed sugars, mainly galactose. LA concentration reached 90.95 g·L^−1^ after 29 h of fermentation and 92.08 g·L^−1^ in the second experiment, after 52 h. 

During the fermentation with molasses as substrate, the difference between initial sugar concentrations exceeded 10 g·L^−1^. In both cases, there were unconsumed sugars with an amount of 1.58 g·L^−1^ in the first fermentation and 11 g·L^−1^ in the second one, while both fermentations lasted for about 50 h. LA final titre was 93.80 g·L^−1^ and 87.70 g·L^−1^, respectively. The distribution in sugars concentration in both cases of sugar bread fermentations had a similar behaviour with final LA concentrations of 80.0 g·L^−1^ and 77.84 g·L^−1^ after almost 30 h of fermentation. In both fermentations, there were almost 2 g·L^−1^ of unconsumed sugars (Figure 1e,f).

Tapioca starch hydrolysate contained a very high initial glucose concentration (almost 140 g·L^−1^), which most probably caused a stress to the strain, as lactic acid production was initiated after 8 h of fermentation, for both first and second fermentation (Figure 1g,h). After 40 h, glucose was completely depleted, but 2.4 g·L^−1^ of disaccharides were left unconsumed in both cases. The fermentation profile was very similar, with final lactic acid concentrations of 96.64 g·L^−1^ and 95.26 g·L^−1^, respectively, while a yield of 0.90 g·g^−1^ and a productivity of 2.2 g·L^−1^·h^−1^ was achieved (Table 3).

Additionally, reference fermentation with commercial sugars was performed, where the fermentation of a mixture of glucose, lactose, galactose, sucrose, maltose and arabinose was monitored (Figure 1i). In this case, glucose consumption first occurred with a complete depletion after only 3 h of fermentation. The second most consumed sugar was arabinose, depleted after 6 h of fermentation. The consumption of lactose/sucrose/maltose and galactose was delayed and started after 3 h of fermentation. The fermentation was stopped after 24 h since sugar consumption rate and LA production rate were very slow. Finally, 4.19 g·L^−1^ of lactose/sucrose/maltose and 4.69 g·L^−1^ of galactose remained unconsumed. LA production was rapid during the first 10 h of fermentation and reached 51.5 g·L^−1^ after 24 h of fermentation.

### 3.3. Continuous Fermentations

**Figure 2:**
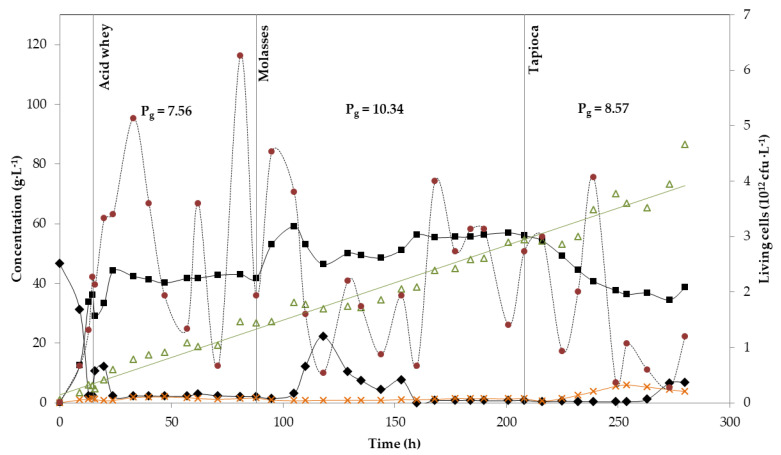
Continuous fermentation using acid whey, molasses and tapioca with *B. coagulans* A534 strain. Concentration in g·L^−1^ of: Total sugars (diamonds), lactic acid (squares), acetic acid (crosses) and cell biomass (triangles). The number of and living cells in cfu per L (circles). Global productivity P_g_ is also shown for each substrate, as g·L^−1^.

**Figure 3:**
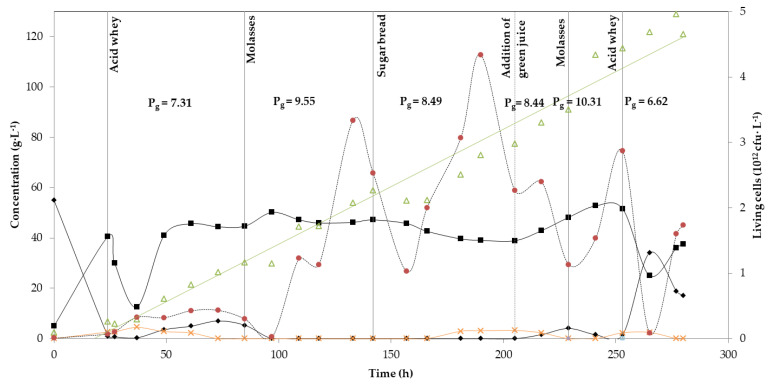
Continuous fermentation using acid whey, molasses, sugar bread and alfalfa green juice with *B. coagulans* A534 strain. Concentration in g·L^−1^ of total sugars (diamonds), lactic acid (squares), acetic acid (crosses) and cell biomass (triangles). Number of living cells in cfu per L (circles). Global productivity P_g_ is also shown for each substrate, as g·L^−1^.

Two continuous lactic acid fermentations were carried out for 280 h, at a constant dilution rate of 0.2 h^−1^. Acid whey was used as the starting substrate for both experiments (Figure 2 and Figure 3). Figure 2 presents the kinetics of the first experiment in which three substrates were shifted in the course of the fermentation (acid whey, molasses and tapioca starch hydrolysate). The batch phase started with 46.7 g·L^−1^ initial lactose concentration and lasted for approximately 15 h, when the residual sugars (lactose) were 2.2 g·L^−1^, while 36.2 g·L^−1^ of lactic acid were produced. Small amounts of acetic acid (1.3 g·L^−1^) were also detected (Figure 2). Acid whey supply continued for 73 h more, before changing the substrate to molasses. During this period, the average LA concentration was 40.1 g·L^−1^ with a yield and productivity of 0.74 g·g^−1^ and 7.56 g·L^−1^·h^−1^, respectively. After ~24 h of fermentation, the residual lactose concentration in the bioreactor was kept at ~2 g·L^−1^, while neither glucose nor galactose were detected (Figure 2), a value that kept stable until changing the substrate from whey to molasses. At the same time, acetic acid production kept also constant at an average concentration of 1.4 g·L^−1^. Acetic acid production is not untypical for *B. coagulans* strains [33,34].

After 88 h (total fermentation time), the substrate was changed to molasses, which was followed by a slight increase in LA concentration to 54 g·L^−1^ (average value). After 22 h of feeding with molasses, sugars started accumulating in the bioreactor and LA production dropped to 47.8 g·L^−1^ (average value). This sudden decrease in strain’s performance could be attributed to the clogging of the filter module due to solids—or even cell biomass—accumulation, as, after changing the membranes, productivity increased again (54.7 g·L^−1^ average LA concentration). The effect of the cell retention system on strain’s performance could also be noticed following the trend of living cells (Figure 2). After ~100 h of fermentation, living cells presented a value of 4.5 × 10^12^ cells· L^−1^ that slowly dropped to 0.5 × 10^12^ cells·L^−1^, to increase again to an average value of 3 × 10^12^ cells·L^−1^. Even with this problem, the overall yield and productivity during this phase was higher in comparison to the acid whey feeding phase (0.86 g·g^−1^ and 10.34 g·L^−1^·h^−1^, respectively).

Changing the substrate from molasses to tapioca (after 208 h of fermentation) resulted in a reduction of LA’s concentration even though no residual sugars were detected. When glucose concentration became limiting, acetic acid formation increased, reaching values of 5 g·L^−1^. LA yield and productivity for this period were 0.70 g·g^−1^ and 8.57 g·L^−1^·h^−1^, respectively. The fermentation was stopped after operating for a total of 280 h, as sugars started accumulating in the bioreactor and the number of living cells dropped below 1 × 10^12^ cells·L^−1^ (Figure 2).

Three different substrates were investigated in the second continuous experiment (acid whey, molasses and sugar bread hydrolysate), and green juice as cheaper alternative to yeast extract, which were changed during the fermentation for five times (Figure 3). Acid whey was again the initial substrate, starting with a lactose concentration of almost 53 g·L^−1^, similar to the first continuous fermentation. In this case, the batch phase lasted for 24 h, resulting in the production of 40.5 g·L^−1^ LA. Lactose was almost consumed at the end of the batch phase (Figure 3). Acid whey was continuously fed in the bioreactor for the next 61 h, during which the average LA concentration was 38.1 g·L^−1^. Sugar concentration was kept at values of 3–5 g·L^−1^ throughout this period. Overall yield and productivity were 0.66 g·g^−1^ and 7.31 g·L^−1^·h^−1^, respectively. Acetic acid production was again observed from the end of the batch phase, with a value of 2.6 g·L^−1^, but after 60 h of fermentation it was completely consumed. Then, after 85 h of fermentation, acid whey was changed to molasses. For the next 57 h, no residual sugars were detected, while LA concentration had an average value of 48 g·L^−1^, with a yield of 0.76 g·g^−1^ and an overall productivity of 9.55 g·L^−1^·h^−1^.

Sugar bread was applied after 142 h of fermentation. The system’s stability was not affected as all sugars were constantly consumed and LA average value was maintained at similar levels as in the previous stage (42 g·L^−1^). However, after ~30 h of using sugar bread hydrolysate as substrate, the strain started to produce both acetic and formic acid, at concentrations of approximately 3 g·L^−1^ and 5 g·L^−1^ respectively. After 205 h, yeast extract was replaced by green juice and by-product formation ceased again. Sugar bread was still the carbon source, and the feeding ratio was kept the same (50:50). Average unconsumed sugar concentration was only 1.5 g·L^−1^, while the average LA concentration was 43.4 g·L^−1^. LA productivity presented similar values to the sugar bread hydrolysate: yeast extract phase (8.44 g·L^−1^·h^−1^), even though yield was a bit lower (0.68 g·g^−1^). After 229 h again molasses was added and at the end, after 253 h of fermentation, acid whey was supplemented. This time, LA productivity was a bit higher (10.31 g·L^−1^·h^−1^), while yield remained almost the same (0.78 g·g^−1^). Acid whey supplementation at the end of experiment led to an increased yield (0.87 g·g^−1^) in comparison to the initial phase, productivity was however lower (6.62 g·L^−1^·h^−1^). Lactose started accumulating in the bioreactor, so the experiment was stopped after 280 h. 

In both cases the only by-product formed was acetic acid, at a concentration <6 g·L^−1^, while the highest productivities were observed when molasses were consumed and reached 10.34 g·L^−1^·h^−1^ and 10.31 g·L^−1^·h^−1^ for the first and second continuous fermentation, respectively. At the same time, yields were 0.86 g·g^−1^ and 0.78 g·g^−1^. The lowest productivity was observed when acid whey was fed in the bioreactor, with values of 7.56 g·L^−1^·h^−1^ for the first and 6.62 g·L^−1^·h^−1^ for the second continuous fermentation. LA yields were 0.74 g·g^−1^ and 0.87 g·g^−1^, respectively. The continuous fermentations resulted in a progressive increase of biomass up to 86.56 g·L^−1^ and 120.92 g·L^−1^ DCW. Additionally, the number of living cells was shown, which could be correlated with the substrate and productivity. The fluctuation in living cells number was higher in the case of the second continuous fermentation, whereas in the first continuous fermentation remained more constant, most probably due to reduced substrate switch. The summary of yields and productivities calculated for each substrate used in both continuous fermentations is shown in Table 4.

## 4. Discussion

The batch experiments carried out with various renewable substrates demonstrate the feasibility to produce high amounts of LA from different raw materials. The ability of *B. coagulans* strains to consume a wide variety of carbon sources and to produce LA with high optical purity (Table 2 and Table 3) gives a huge advantage over other LAB strains. Among the different substrates investigated, tapioca starch hydrolysate resulted in both highest LA final concentrations (>90 g·L^−1^) and yields (0.90 g·g^−1^). Interestingly, even though sugar bread was also a starch-based substrate, the high content of disaccharides could be possibly the reason for the lower yields (Table 3). Starch-based hydrolysates have been proven efficient raw materials for LA production from different strains. In the study of Alexandri et al. [27], defatted rice bran hydrolysates were evaluated as potential fermentation substrate for LA production using a *B. coagulans* hydrolysate. The authors reported a 75.9 g·L^−1^ final LA concentration with yield and productivity equal to 0.90 g·g^−1^ and 2.7 g·L^−1^·h^−1^, respectively. The work of Kwan et al. [35] focused on the utilisation of mixed food and bakery wastes for LA production with the strain *Lactobacillus casei* Shirota. The authors achieved final LA titers of 94 g·L^−1^ and 82.6 g·L^−1^ from food and bakery wastes, respectively, while yield was 0.94 g·g^−1^ for both media. A LA concentration of 198.32 g·L^−1^ was obtained from the fermentation of raw sweet potato, enriched with yeast extract and peptone, with *Lactobacillus paracasei*, in a simultaneous saccharification and fermentation strategy [36].

*B. coagulans* was also able to metabolise lactose directly, eliminating the need for prior hydrolysis. Even though productivities were different between the two batches, final lactic acid titres and yield were very similar, indicating a rather efficient bioprocess (Table 3). The obtained final concentrations (>90 g·L^−1^) are amongst the highest in the literature. Panesar et al. [37] reported the production of 33.73 g·L^−1^ L-LA using *L. casei*, while Taleghani et al. [38] reported a value of 32.1 g·L^−1^ when whey was fermented by *L. bulgaricus*. Molasses derived either from sugarcane or sugar beets fermentation to lactic acid has also been investigated, using various LAB strains [39,40,41]. In the recent work of Sun et al. [41], a microbial consortium in which *Clostridium sensu stricto* was the predominant strain was tested for sugarcane molasses fermentation to lactic acid. Even though 112.34 g·L^−1^ of lactic acid were produced, optical purity was 95.6%.

LA optical purity was very high in all the investigated substrates (Table 2). The lowest value was observed in the fermentation with molasses (98%). Molasses contained a high initial amount of LA (7 g·L^−1^), which was a racemic mixture of D- and L-LA, being partly responsible for the lower purities.

The only substrate that was used in continuous fermentation but not for the batch experiments was alfalfa green juice, as it has been previously demonstrated as efficient source of nutrients for LA production [26]. It should be noted that lactic acid producing strains require complex nitrogen sources, such as yeast extract and peptone, which have been proven to improve lactic acid production [36,42]. Yeast extract is rich in vitamin B and essential amino acids that promote cell growth, however it considerably increases the cost of lactic acid production, which can account as high as the 38% of total upstream cost [9,26]. For this reason, many researchers have tried to evaluate alternative—and cheaper—nitrogen sources. Some examples include protein isolate from defatted rice bran [43], defatted algal biomass [44], or the protein present in food waste [45] to name a few. Finally, in the work of Dietz et al. [26] alfalfa green juices from different harvesting periods were used as fermentation substrates for LA production with a *B. coagulans* isolate. The authors reported final LA concentrations in the range of 90 to 99 g/L, with a yield of more than 0.80 g·g^−1^, while the L-LA optical purity was higher than 99.8%. 

Many studies have highlighted the advantage of continuous fermentation mode on LA productivity, despite the substrate used [10,33]. The higher productivities are mainly attributed to lower product inhibition, since LA is constantly removed from the bioreactor. At the same time, operating with cell retention, high cell densities can be achieved, leading to a more stable system and subsequently higher yields and productivities [33]. Ahring et al. [10] obtained the highest LA productivity (3.69 g·L^−1^·h^−1^) in a continuous fermentation with corn stover hydrolysate, operating at a dilution rate of 0.167 h^−1^. Wee et al. [46] reported a 1.6-fold productivity increment when a cell recycle system was employed for the continuous fermentation of lignocellulosic hydrolysates. The hydrolysates were enriched with corn steep liquor and yeast extract. The authors achieved 6.7 g·L^−1^·h^−1^ lactic acid productivity operating at a dilution rate of 0.16 h^−1^. 

In a recent publication from our group [33], continuous fermentations using tapioca starch hydrolysate, acid whey and sugar beet molasses were presented as case studies. Two *B. coagulans* isolates (A107 and A40) were selected for the continuous fermentations with tapioca starch hydrolysate and molasses. When tapioca starch hydrolysate was employed, similar LA average concentration was achieved (50.3 g·L^−1^), but higher yield and productivity were reported (0.80 g·g^−1^ and 10.11 g·L^−1^·h^−1^, respectively). This can be explained by the quick transition to tapioca from molasses. From Figure 3, it is evident that sugar consumption rate was not yet stabilised. Regarding molasses fermentation, higher productivities were achieved in this study (9.55 g·L^−1^·h^−1^ and 10.34 g·L^−1^·h^−1^), in comparison to the one reported (4.89 g·L^−1^·h^−1^) by López-Gómez et al. [33]. This could be attributed to variations on initial sugar content of molasses, as well as the different dilution rate which was 0.1 h^−1^ in comparison to 0.2 h^−1^ used in this study. Increasing the dilution rate can lead to higher LA productivities especially when a cell retention system is used.

By-product formation was observed during some phases in both continuous experiments as well as during the batch cultures. *B. coagulans* strains are homofermentative and L-lactic acid is the main product of their metabolism [21]. Hexoses are metabolised through the Embden–Meyerhof–Parnas (EMP) pathway, which efficiently leads to lactic acid production. Acetate and succinate can also be produced, while formation of formate and acetate were associated to glucose starvation in lactic acid bacteria [4,21]. Similar observations have been previously reported when a *B. coagulans* isolate was employed [33,34].

Another important finding is that the substitution of yeast extract to green juice did not negatively affect fermentation’s performance (Figure 3). The importance of yeast extract for the growth of lactic acid bacteria was stated above, but Payot et al. [34] had emphasised its significance on *B. coagulans* performance. The authors noticed that the highest values of biomass and lactic acid production were attained by yeast extract addition to the fermentation medium. From Figure 3 we cannot safely conclude that green juice had a negative of positive effect on cell biomass, but it is evident that lactic acid production was not reduced. More experiments with green juice supplementation would elucidate the role of this alternative nitrogen source on lactic acid production.

## 5. Conclusions

LA production was evaluated on four inexpensive feedstocks in batch mode in order to assess the performance of *B. coagulans* isolate. The strain was able to efficiently produce LA utilising the sugars from all substrates, resulting in higher yields even in comparison to reference fermentation with commercial sugars. Two continuous fermentations were subsequently carried out switching different substrates in the course of the process. Operating with a cell retention system, high cell densities were achieved, leading to high productivities regardless the substrate fed in the bioreactor. The highest productivities were achieved when molasses were employed with a value of almost 10 g·L^−1^·h^−1^, which is, to the best of our knowledge, the highest for LA continuous fermentation so far. This study demonstrated that LA can be efficiently produced in continuous mode regardless the substrate, which is a huge advantage in comparison to other platform chemicals. 

## Figures and Tables

**Table 1:** Composition of substrates before pretreatment.

Component	Acid Whey	Molasses (240 g·L^−1^)	Sugar Bread (SB)	Alfalfa Juice	Tapioca (% DW)
Glucose (g·L^−1^)	BDL	56.6	3.96	4.57	13.81% DW (Starch 95.02% DW)
Fructose (g·L^−1^)	14.48	17.0	0.90	4.68	
Galactose (g·L^−1^)	BDL	BDL	BDL	BDL	n.d.
Disaccharides (g·L^−1^)	397	505	22.91	1.42	n.d.
Lactose (g·L^−1^)	419	BDL	BDL	BDL	n.d.
Lactic acid (g·L^−1^)	30.71	29.7	BDL	BDL	n.d.
Total nitrogen (mg·L^−1^)	8370.00	18,922	14,514 ^a^	2078	0.022% DW
Total phosphorus (mg·L^−1^)	8525.00	39.5	1863 ^a^	220	n.d.
Cl^−^ (mg·L^−1^)	54,522	1766	n.d.	672	n.d.
SO_4_^2+^ (mg·L^−1^)	6475	7177	n.d.	906	n.d.
Na^+^ (mg·L^−1^)	28,414	10,258	n.d.	26.5	n.d.
K^+^ (mg·L^−1^)	56,456	36,048	n.d.	2809	n.d.
Mg^2+^ (mg·L^−1^)	1273	38.6	n.d.	233	n.d.
Ca^2+^ (mg·L^−1^)	1910	1061	n.d.	899	n.d.

^a^ mg·kg^−1^; BDL—below detection limit; n.d.—not detected.

**Table 2:** Composition of substrates after pretreatment.

Component	Acid Whey Microfilter. 0.2 µm, Diluted, Autoclaved at 121 °C	Molasses Diluted and Sterilised at 121 °C	SB Hydrolysate	Alfalfa Juice Microfilter. 0.2 µm	Tapioca Hydrolysate	Yeast Extract
Glucose (g·L^−1^)	0.75	12.2	76.9	4.60	141.6	BDL
Fructose (g·L^−1^)	n.d.	10.7	9.98	10.3		BDL
Galactose (g·L^−1^)	5.44	n.d.	n.d.	n.d.	n.d.	n.d.
Disaccharides (g·L^−1^)	n.d.	109 (sucrose)	38.5 (sucrose)	n.d.	3.34	1.08 ^a^
Lactose (g·L^−1^)	122	n.d.	n.d.	n.d.	n.d.	n.d.
Lactic acid (g·L^−1^)	7.62	6.34	n.d.	n.d.	n.d.	n.d.
Total nitrogen (mg·L^−1^)	1233	4516	193	1086	25	106.7 ^b^
Total phosphorus (mg·L^−1^)	2601	32	164	463	36	15.7 ^b^
Cl^−^ (mg·L^−1^)	12,411	479	175	632	23	n.d.
SO_4_^2+^ (mg·L^−1^)	1261.0	1718.0	73.4	398.5	25.1	n.d.
Na^+^ (mg·L^−1^)	5134	2121	318	22	44	3.11 ^b^
K^+^ (mg·L^−1^)	10,600	7821	225	2619	50	31.33 ^b^
Mg^2+^ (mg·L^−1^)	255.4	9.5	27.2	170.6	5.2	1.58 ^b^
Ca^2+^ (mg·L^−1^)	537.7	227.1	72.3	507.2	9.5	0.57 ^b^
Total Carbon (g·L^−1^)	56.89	34.64	50.96	5.96	58.04	0.45

^a^ % DW; ^b^ g·kg^−1^; BDL—below detection limit; n.d.—not detected.

**Table 3:** Yields and productivities calculated for batch fermentations with *B. coagulans* A534 strain.

Substrate	Total Sugars (g·L^−1^)	Yield (g·g^−1^)	P_g_ (g·L^−1^·h^−1^)	P_max_ (g·L^−1^·h^−1^)	LA (g·L^−1^)	LA Optical Purity (%)
Acid whey (a)	107.30	0.85	2.84	5.72	90.95	99.2
Acid whey (b)	107.94	0.85	1.77	4.93	92.08	99.1
Molasses (c)	143.39	0.88	2.00	7.62	93.80	98.0
Molasses (d)	132.37	0.90	1.89	4.13	87.70	98.0
Sugar bread (e)	120.41	0.85	2.67	9.26	80.00	99.6
Sugar bread (f)	119.50	0.74	3.00	8.86	77.84	99.6
Tapioca (g)	140.45	0.93	2.25	6.57	96.64	n.d.
Tapioca (h)	139.12	0.90	2.22	6.70	95.26	n.d.
Sugar mix (i)	78.90	0.79	2.15	9.28	51.51	99.8

n.d.—not detected.

**Table 4:** Yield and global productivities calculated for two continuous fermentations.

Parameter	Acid Whey	Molasses	Sugar Bread	Sugar Bread: Green Juice	Molasses	Acid Whey	Tapioca
P_g_ (g·L^−1^·h^−1^)	7.56 */7.31	10.34 */9.55	8.49	8.44	10.31	6.62	8.57 *
Yield (g·g^−1^)	0.74 */0.66	0.86 */0.76	0.76	0.68	0.78	0.87	0.70 *

* Continuous fermentation 1.

## References

[1] Grand View Research (2019). Lactic Acid Market Size, Share & Trends Analysis Report By Raw Material (Sugarcane, Corn, Cassava), by Application (Industrial, F&B, Pharmaceuticals, Personal Care, PLA), And Segment Forecasts, 2018–2025.

[2] Ioannidou S.M., Pateraki C., Ladakis D., Tsakona M., Vlysidis A., Kookos I.K. (2020). Sustainable production of bio-based chemicals and polymers via integrated biomass refining and bioprocessing in a circular bioeconomy context. Bioresour. Technol..

[3] González M.I., Álvarez S., Riera F., Álvarez R. (2007). Economic evaluation of an integrated process for lactic acid production from ultrafiltered whey. J. Food Eng..

[4] Alves de Oliveira R., Komesu A., Vaz Rossell C.E., Maciel Filho R. (2018). Challenges and opportunities in lactic acid bioprocess design—From economic to production aspects. Biochem. Eng. J..

[5] Cubas-Cano E., González-Fernández C., Ballesteros I., Tomás-Pejó E. (2020). Efficient utilization of hydrolysates from steam-exploded gardening residues for lactic acid production by optimization of enzyme addition and pH control. Waste Manag..

[6] Pleissner D., Demichelis F., Mariano S., Fiore S., Schneider R., Venus J., Michelle I., Guti N. (2017). Direct production of lactic acid based on simultaneous sacchari fi cation and fermentation of mixed restaurant food waste. J. Clean. Prod..

[7] López-Gómez J.P., Unger P., Schneider R., Venus J. (2020). From Upstream to Purification: Production of Lactic Acid from the Organic Fraction of Municipal Solid Waste. Waste Biomass Valorization.

[8] López-Gómez J.P., Latorre-Sánchez M., Unger P., Schneider R., Coll Lozano C., Venus J., Lozano C.C., Venus J. (2019). Assessing the organic fraction of municipal solid wastes for the production of lactic acid. Biochem. Eng. J..

[9] Alves de Oliveira R., Schneider R., Lunelli B.H., Rossell C.E.V., Filho R.M., Venus J. (2020). A Simple Biorefinery Concept to Produce 2G-Lactic Acid from Sugar Beet Pulp (SBP): A High-Value Target Approach to Valorize a Waste Stream. Molecules.

[10] Ahring B.K., Traverso J.J., Murali N., Srinivas K. (2016). Continuous fermentation of clarified corn stover hydrolysate for the production of lactic acid at high yield and productivity. Biochem. Eng. J..

[11] Putra M.D., Abasaeed A.E. (2018). A more generalized kinetic model for binary substrates fermentations. Process Biochem..

[12] Calderon M., Loiseau G., Guyot J.P. (2003). Fermentation by Lactobacillus fermentum Ogi E1 of different combinations of carbohydrates occurring naturally in cereals: Consequences on growth energetics and α-amylase production. Int. J. Food Microbiol..

[13] Kwon Y.J., Engler C.R. (2005). Kinetic models for growth and product formation on multiple substrates. Biotechnol. Bioprocess. Eng..

[14] Burgos-Rubio C.N., Okos M.R., Wankat P.C. (2000). Kinetic Study of the Conversion of Different Substrates to Lactic Acid Using Lactobacillus bulgaricus. Biotechnol. Prog..

[15] Lu H., Zhao X., Wang Y., Ding X., Wang J., Garza E., Manow R., Iverson A., Zhou S. (2016). Enhancement of D-lactic acid production from a mixed glucose and xylose substrate by the Escherichia coli strain JH15 devoid of the glucose effect. BMC Biotechnol..

[16] Zabaniotou A. (2018). Redesigning a bioenergy sector in EU in the transition to circular waste-based Bioeconomy—A multidisciplinary review. J. Clean. Prod..

[17] Bonk F., Bastidas-Oyanedel J.R., Yousef A.F., Schmidt J.E., Bonk F. (2017). Exploring the selective lactic acid production from food waste in uncontrolled pH mixed culture fermentations using different reactor configurations. Bioresour. Technol..

[18] Tashiro Y., Inokuchi S., Poudel P., Okugawa Y., Miyamoto H., Miayamoto H., Sakai K. (2016). Novel pH control strategy for efficient production of optically active l-lactic acid from kitchen refuse using a mixed culture system. Bioresour. Technol..

[19] Peinemann J.C., Rhee C., Shin S.G., Pleissner D. (2020). Non-sterile fermentation of food waste with indigenous consortium and yeast–Effects on microbial community and product spectrum. Bioresour. Technol..

[20] Tang J., Wang X., Hu Y., Zhang Y., Li Y. (2016). Lactic acid fermentation from food waste with indigenous microbiota: Effects of pH, temperature and high OLR. Waste Manag..

[21] Su F., Xu P. (2014). Genomic analysis of thermophilic Bacillus coagulans strains: Efficient producers for platform bio-chemicals. Sci. Rep..

[22] Aulitto M., Fusco S., Bartolucci S., Franzén C.J., Contursi P. (2017). *Bacillus coagulans* MA-13: A promising thermophilic and cellulolytic strain for the production of lactic acid from lignocellulosic hydrolysate. Biotechnol. Biofuels.

[23] Alves de Oliveira R., Schneider R., Vaz Rossell C.E., Maciel Filho R., Venus J. (2019). Polymer grade l-lactic acid production from sugarcane bagasse hemicellulosic hydrolysate using Bacillus coagulans. Bioresour. Technol. Rep..

[24] Sakai K., Ezaki Y. (2006). Open L-lactic acid fermentation of food refuse using thermophilic *Bacillus coagulans* and fluorescence in situ hybridization analysis of microflora. J. Biosci. Bioeng..

[25] Pleissner D., Neu A.K., Mehlmann K., Schneider R., Puerta-Quintero G.I., Venus J. (2016). Fermentative lactic acid production from coffee pulp hydrolysate using Bacillus coagulans at laboratory and pilot scales. Bioresour. Technol..

[26] Dietz D., Schneider R., Papendiek F., Venus J. (2016). Leguminose green juice as an efficient nutrient for L(+)-lactic acid production. J. Biotechnol..

[27] Alexandri M., Neu A., Schneider R., Pablo L., Venus J. (2018). Evaluation of various Bacillus coagulans isolates for the production of high purity L-lactic acid using defatted rice bran hydrolysates. Int. J. Food Sci. Technol..

[28] Abdel-Banat B.M.A., Hoshida H., Ano A., Nonklang S., Akada R. (2010). High-temperature fermentation: How can processes for ethanol production at high temperatures become superior to the traditional process using mesophilic yeast?. Appl. Microbiol. Biotechnol..

[29] Olszewska-Widdrat A., Alexandri M., López-Gómez J.P., Schneider R., Mandl M., Venus J. (2019). Production and Purification of l-lactic Acid in Lab and Pilot Scales Using Sweet Sorghum Juice. Fermentation.

[30] ISO 15681-1 (2003). Water Quality—Determination of Orthophosphate and Total Phosphorus Contents by Flow Analysis (FIA and CFA)—Part 1: Method by Flow Injection Analysis (FIA) Qualité.

[31] Chandrapala J., Duke M.C., Gray S.R., Weeks M., Palmer M., Vasiljevic T. (2017). Strategies for maximizing removal of lactic acid from acid whey–Addressing the un-processability issue. Sep. Purif. Technol..

[32] Chen G.Q., Eschbach F.I.I., Weeks M., Gras S.L., Kentish S.E. (2016). Removal of lactic acid from acid whey using electrodialysis. Sep. Purif. Technol..

[33] López-Gómez J.P., Alexandri M., Schneider R., Venus J. (2019). A review on the current developments in continuous lactic acid fermentations and case studies utilising inexpensive raw materials. Process Biochem..

[34] Payot T., Chemaly Z., Fick M. (1999). Lactic acid production by Bacillus coagulans-Kinetic studies and optimization of culture medium for batch and continuous fermentations. Enzyme Microb. Technol..

[35] Kwan T.H., Hu Y., Lin C.S.K. (2016). Valorisation of food waste via fungal hydrolysis and lactic acid fermentation with *Lactobacillus casei* Shirota. Bioresour. Technol..

[36] Nguyen C.M., Choi G.J., Choi Y.H., Jang K.S., Kim J.C. (2013). D- and l-lactic acid production from fresh sweet potato through simultaneous saccharification and fermentation. Biochem. Eng. J..

[37] Panesar P.S., Kennedy J.F., Knill C.J., Kosseva M., Patel S.A., Parikh S.C. (2010). Production of L(+) Lactic Acid using Lactobacillus casei from Whey. Int. J. Curr. Microbiol. Appl. Sci..

[38] Taleghani H.G., Najafpour G.D., Ghoreyshi A.A. (2016). A study on the effect of parameters on lactic acid production from whey. Pol. J. Chem. Technol..

[39] Mladenović D.D., Djukić-Vuković A.P., Kocić-Tanackov S.D., Pejin J.D., Mojović L.V. (2016). Lactic acid production on a combined distillery stillage and sugar beet molasses substrate. J. Chem. Technol. Biotechnol..

[40] Abdel-Rahman M.A., Tan J., Tashiro Y., Zendo T., Sakai K., Sonomoto K. (2020). Non-carbon loss long-term continuous lactic acid production from mixed sugars using thermophilic Enterococcus faecium QU 50. Biotechnol. Bioeng..

[41] Sun Y., Xu Z., Zheng Y., Zhou J., Xiu Z. (2019). Efficient production of lactic acid from sugarcane molasses by a newly microbial consortium CEE-DL15. Process Biochem..

[42] Klotz S., Kuenz A., Prüße U. (2017). Nutritional requirements and the impact of yeast extract on the d-lactic acid production by Sporolactobacillus inulinus. Green Chem..

[43] Wang Y., Yang Z., Qin P., Tan T. (2014). Fermentative l-(+)-lactic acid production from defatted rice bran. RSC Adv..

[44] Pleissner D., Lau Y., Zhang C., Sze C., Lin K. (2015). Plasticizer and Surfactant Formation from Food-Waste- and Algal Biomass-Derived Lipids. ChemSusChem.

[45] Peinemann J.C., Demichelis F., Fiore S., Pleissner D. (2019). Techno-economic assessment of non-sterile batch and continuous production of lactic acid from food waste. Bioresour. Technol..

[46] Wee Y.J., Ryu H.W. (2009). Lactic acid production by Lactobacillus sp. RKY2 in a cell-recycle continuous fermentation using lignocellulosic hydrolyzates as inexpensive raw materials. Bioresour. Technol..

